# Thromboelastographic Assessment of Coagulation Profiles in Dogs with Cardiac Tumors and Their Relationship to Cardiac Function

**DOI:** 10.3390/ani15182674

**Published:** 2025-09-12

**Authors:** Zeki Yilmaz, Hakan Salci, Pınar Levent, Didem Algan, Tuğba Varlik, Mehmet Emre Topçu, Ryou Tanaka, Lina Hamabe

**Affiliations:** 1 Department of Internal Medicine, Veterinary Faculty, Bursa Uludag University, 16059 Bursa, Turkey; zyilmaz@uludag.edu.tr (Z.Y.); vetpinarlevent@gmail.com (P.L.); didem.algann@gmail.com (D.A.); tugbavarlik777@gmail.com (T.V.); 2 Department of Surgery, Veterinary Faculty, Bursa Uludag University, 16059 Bursa, Turkey; hsalci@uludag.edu.tr; 3 VetPlanet Veterinary Clinic, 16140, Bursa, Turkey; 4 Department of Pathology, Veterinary Faculty, Bursa Uludag University, 16059 Bursa, Turkey; mehmetemretopcu@uludag.edu.tr; 5 Life Science, Hamebe Team, Institute of Global Innovation Research, Tokyo University of Agriculture and Technology, Tokyo 183-8538, Japan; ryo@vet.ne.jp

**Keywords:** onco-cardiology, coagulation, global clotting time, thromboelastography, dogs

## Abstract

Cardiac tumors are rare and linked to high mortality rates in both human and veterinary medicine. Despite their clinical significance, the effects of these tumors on coagulation and cardiac function remain poorly understood. This retrospective study assessed coagulation profiles and echocardiographic parameters in 14 dogs with cardiac tumors compared to 10 healthy controls. Tumors were identified through echocardiography, with further confirmation by computed tomography/magnetic resonance imaging. Coagulation was evaluated using conventional tests (prothrombin time (PT) and activated partial thromboplastin time (aPTT)) and thromboelastography (TEG). The employed conventional coagulation tests and echocardiographic parameters showed no significant differences between the groups. However, TEG revealed a hypercoagulable state in the majority of tumor-bearing dogs (8 out of 14), characterized by shortened reaction and clot formation times, as well as an increased α-angle and coagulation index (*p* < 0.05–0.001). No significant correlations were found between TEG parameters and echocardiographic indices. This study is the first to demonstrate subclinical hemostatic alterations using TEG in dogs with cardiac neoplasia. The results support the utility of TEG as a more sensitive tool than conventional tests for detecting hypercoagulability, potentially guiding individualized anticoagulant strategies in affected dogs.

## Simple Summary

Cardiac tumors are serious conditions that can impair both cardiac function and the coagulation system, potentially leading to hemorrhagic or thrombotic complications. In this study, we examined the impact of cardiac tumors on hemostasis in dogs, aiming to elucidate the associated coagulation changes and assess cardiac structure and function. Standard coagulation assays, including prothrombin time (PT) and activated partial thromboplastin time (aPTT), were employed alongside thromboelastography (TEG), an advanced diagnostic tool that provides a comprehensive evaluation of clot formation and fibrinolysis. While PT and aPTT did not demonstrate significant changes between the groups, TEG parameters revealed notable alterations in hemostatic profiles among dogs with cardiac tumors. Echocardiographic measurements showed no significant differences in cardiac chamber size and function between healthy dogs and those with tumors. To the best of our knowledge, this is the first study to evaluate hemostatic alterations in dogs with cardiac tumors using TEG. These findings suggest that TEG may offer superior sensitivity compared to conventional tests for detecting hemostatic abnormalities in dogs with cardiac neoplasia. An enhanced understanding of these coagulation changes may support improved diagnostic assessments and inform therapeutic strategies in veterinary cardiology.

## 1. Introduction

Cardiac tumors in dogs, although relatively uncommon, pose significant diagnostic and therapeutic challenges due to their insidious clinical presentation and potential for life-threatening complications such as pericardial effusion, cardiac tamponade, arrhythmias, and thromboembolic events [1,2,3,4,5]. Aortic body tumors are the most frequent and are most commonly represented by chemodectomas. Hemangiosarcomas, however, are not considered aortic body tumors and are usually localized to the right atrium/auricle, often associated with pericardial effusion [1,2,6].

Tumor biology and hemostasis are intricately interlinked; malignancies may promote coagulation through multiple mechanisms, including the release of procoagulant microparticles, inflammatory cytokines, and direct endothelial injury. Conversely, components of the coagulation system can influence tumor progression, angiogenesis, and metastatic potential [7,8]. As a result, patients with tumors frequently exhibit coagulation disturbances, manifesting as either bleeding tendencies or thrombotic events, depending on tumor type, stage, and individual susceptibility [9]. Despite this mechanistic plausibility, the systemic effects of cardiac tumors on coagulation and their potential interplay with cardiac structure and function remain poorly understood.

In dogs with cardiac tumors, the hemostatic balance may be disrupted by tumor-associated procoagulant activity, inflammatory cytokine release, and endothelial damage, potentially predisposing affected animals to bleeding diatheses or hypercoagulable states [10,11]. Traditional plasma-based coagulation tests such as prothrombin time (PT) and activated partial thromboplastin time (aPTT) provide limited insight into the overall functionality of the hemostatic system, as they only evaluate discrete components of the coagulation cascade under static conditions [12,13].

Thromboelastography (TEG), a viscoelastic whole-blood assay, offers a comprehensive and dynamic assessment of clot formation, strength, and fibrinolysis, thus allowing for a more physiologically relevant evaluation of coagulation status in clinical settings [14,15]. TEG has been increasingly utilized in veterinary medicine to detect hypercoagulability in dogs with neoplastic [10], inflammatory [14,16], infectious [17], or cardiovascular disorders [13,16].

Given the potential for both hemorrhagic and thrombotic complications in dogs with cardiac tumors, comprehensive assessment of coagulation profiles using both conventional tests and TEG may yield important mechanistic insights into tumor-associated hemostatic dysregulation. Moreover, aberrations in coagulation may also reflect alterations in cardiac function, further reinforcing the interdependence between the cardiovascular and hemostatic systems [18,19].

The objective of this retrospective study was to evaluate the coagulation profiles and cardiac functions of dogs diagnosed with cardiac tumors, with a particular focus on the utility of TEG and conventional clotting tests in identifying coagulation abnormalities and their potential correlation with echocardiographic findings.

## 2. Materials and Methods

### 2.1. Study Population

This study involved a total of 24 client-owned dogs selected from the archive of the Veterinary Teaching Hospital (Department of Internal Medicine, Veterinary Faculty, Bursa Uludağ University, Bursa, Türkiye) and from private clinics where the first author (Z.Y.) provides official consultancy services: 14 dogs diagnosed with cardiac tumors (CT group) and 10 healthy controls (HC group). The dogs in the CT group consisted of various breeds, different ages, and both sexes. Control dogs were matched to the CT group based on age, breed, and sex. As all diagnostic procedures were part of routine clinical care, institutional ethical approval was obtained for this retrospective study.

### 2.2. Inclusion and Exclusion Criteria

Dogs with cardiac tumors, which were localized by transthoracic echocardiography (Caris Plus and MyLab^TM^ X5Vet, Esoate, Florence, Italy; and Vivid S60 Ultra Edition, EchoPack^TM^ PC Version 204, Oslo, Norway), confirmed using computed tomography (3D, 2 sequence, Siemens^®^, Forchheim, Germany) or magnetic resonance imaging (MRI) (1.5 tesla, General Electric^®^, Irvine, CA, USA), were included in the study. In addition to the echocardiographic definition, fine-needle aspiration (FNA) was performed from pericardial or pleural effusion in most cases, and directly from the mass in selected cases. Dogs with concurrent systemic diseases (e.g., infection or metabolic and endocrine diseases), recent surgeries, or receiving medications affecting coagulation, such as anticoagulants or antiplatelet agents, within the last two weeks were excluded. Furthermore, none of the included animals were obese or exhibited clinical evidence of systemic disease unrelated to cardiac tumors.

Healthy dogs were retrospectively selected from hospital records of dogs presented to the clinic for comprehensive health checks at the owners’ request. Dogs with no clinical, hematological, or echocardiographic abnormalities, and for whom coagulation assays were available, were considered for inclusion in the HC group, as reported previously [20].

### 2.3. Clinical Parameters

At presentation, each dog underwent a complete physical examination. Vital parameters such as heart and respiratory rates, rectal temperature, and body weight were recorded. In some cases, non-invasive blood pressure was measured (Veterinary Vital Sign Monitor, Hasvet, China), as suggested [21].

### 2.4. Hematologic and Serum Biochemical Analysis

Venous blood samples were collected from the cephalic vein under minimal restraint into EDTA-containing vacutainer tubes for hematologic analysis and tubes without anticoagulant for serum biochemistry analysis. Complete blood counts (CBCs) were performed using an automated hematology analyzer (VetScan HM5, Abaxis, Union City, CA, USA), and serum biochemistry panels were analyzed using a clinical chemistry analyzer (VetScan VS2, Abaxis) and included measurements of the hepato-renal injury markers alanine aminotransferase (ALT), alkaline phosphatase (ALP), blood urea nitrogen (BUN), and creatinine.

### 2.5. Coagulation Analyses

Global coagulation tests, including PT and aPTT, were measured using an automated coagulometer (VSPro Veterinary Coagulation Analyzer, Abaxis) [14,16]. For TEG analysis, whole blood samples were collected using a 21-gauge needle into vacutainer tubes containing 3.2% sodium citrate. Samples were analyzed within 30 min of collection using kaolin as the activator on a TEG^®^ 5000 Thromboelastography Hemostasis Analyzer (Haemonetics Corp., Boston, MA, USA). In this study, the kaolin reagent was first restored to room temperature, and then 1.0 mL of a blood sample was added to the reagent. The tube was gently inverted and mixed five times, and 20 μL of 0.2 mol/L calcium chloride was added to the preheated reaction cup of the TEG instrument. Subsequently, 340 μL of the sample was drawn and mixed with kaolin (a total of 360 μL) in the reaction cup. The cup slot was moved up, and the tests were started, as performed in our [14,16] and other studies [22].

The following TEG parameters were recorded to evaluate viscoelastic properties of blood clot formation: reaction time (R) and clot kinetics (K) for initial clot formation; α-angle, maximum amplitude (MA), clot strength (G), and projected maximum amplitude (PMA) for clot strength and stability; A-value, estimated percent lysis (EPL), and lysis at 30 min (LY30) for fibrinolytic activity; and coagulation index (CI), which is an overall estimation of coagulation. All TEG measurements were performed at 37 °C, as previously described [23].

The hypercoagulable state of TEG was defined as 2 or more of a combination of significantly higher α-angle, MA, or G, or lower R or K, compared to the group of healthy control dogs. The hypocoagulable state was defined as 2 or more of a combination of significantly lower α-angle, MA, or G, or higher R or K, compared to the healthy dogs [24].

### 2.6. Echocardiographic Assessment

All dogs without sedation underwent transthoracic echocardiography using a multifrequency phased-array transducer (5–7.5 MHz, Caris Plus or MyLab X5Vet, Esoate, Florence, Italy; or 3 s and 6 s cardiac probes, Vivid S60 Ultra edition, Norway) by an experienced veterinary cardiologist (Z.Y.). Echocardiographic measurements were averaged from three consecutive cardiac cycles obtained during quiet respiration, with the animal in right or left lateral recumbency. Cardiac imaging included 2-dimensional, M-mode, Doppler echocardiography, and 2D speckle tracking echocardiography with right parasternal short- (RPSAx) and long-axis (RPLAx), subcostal, and apical 2-, 3-, and 4-chamber views [25].

Amongst the cardiac measurements, parameters regarding cardiac anatomy and left ventricular systolic function were selected for this study: the left atrium-to-aorta ratio (LA/Ao) from aortic level at RPSAx, and left ventricular internal diameter normalized for body weight (LVIDdN) from M-Mode measurements at papillary muscle levels at RPSAx. Left ventricular systolic function was evaluated by fractional shortening (FS%) and ejection fraction (EF%) using the Teichholz method (M-Mode, papillary muscle level at RPSAx). In one case with an intracardiac tumor, auto-EF and global longitudinal strain (GLS%) were also determined using the Bull’s eye image, as previously described [26,27].

### 2.7. Tumor Diagnosis and Typing

Definitive diagnosis of cardiac tumors in dogs remains challenging due to anatomical constraints. Tumors localized at the heart base were presumptively diagnosed based on echocardiographic findings, anatomical location, clinical presentation, and imaging characteristics, following established criteria [28]. Cardiovascular tumors, except one (aortic body chemodectoma) that could not be sampled for cytological analysis, in all cases were defined as a heart base tumor using transthoracic echocardiography [1,2] and their localizations were confirmed using diagnostic imaging techniques (computed tomography and/or magnetic resonance imaging (MRI)) [29]. Application and evaluation of these techniques were performed by H.S., an experienced veterinary surgeon.

Fine-needle aspiration cytology was performed to type other tumors, including pericardial mesothelioma (n = 4), cardiac hemangiosarcomas (n = 2), aortic body tumors (chemodectoma) (n = 1), and lymphoma (n = 1) [1]. The aspirated material was then carefully expelled onto clean glass slides, allowed to dry at room temperature, and stained using the Diff-Quick method. The prepared smears were examined under a light microscope (Olympus BX51, Tokyo, Japan) at various magnifications (×40–1000, including oil immersion for detailed cellular evaluation), and cytological images were taken. One case involved a large right atrial mass for which cytological confirmation was not obtained, as the owner declined the sampling procedure.

### 2.8. Statistical Analysis

All data were analyzed using statistical software (SigmaPlot v16, GmBH, Germany). Normality of data distribution was evaluated using the Shapiro–Wilk test. Normally distributed continuous data are expressed as the mean ± standard deviation (SD), while non-normally distributed data are reported as the median and minimum–maximum. Group comparisons were performed using the independent samples *t*-test for normally distributed variables. Correlations between coagulation parameters (TEG, PT, and aPTT) and cardiac indices (LA/Ao, LVIDdN, FS%, and EF%) were evaluated using Spearman’s rank correlation coefficient. A *p*-value < 0.05 was considered statistically significant.

## 3. Results

### 3.1. Signalment and Clinical Findings

The dogs selected for this study were of different breeds, including Golden retrievers (n = 5), labrador (n = 2), German shepherd (n = 2), boxer (n = 1), English bulldog (n = 1), cane corso (n = 1), and mixed breed (n = 2) (Table 1 and Table 2). There were no statistically significant differences in demographic data between the groups. Dogs with cardiac tumors showed a significantly higher heart rate (HR; *p* < 0.01) and respiratory rate (RR; *p* < 0.001) compared to controls (Table 1). Among the seven dogs with pericardial and/or pleural effusion, HR and RR tended to be higher compared to those without effusion, although this difference was not statistically significant due to the wide range of both values. In three dogs from the CT group for whom blood pressure measurements were available (Cases 12, 13, and 14), hypertension was detected, with mean arterial pressure values ranging from 162 to 177 mm Hg.

**Figure 1:**
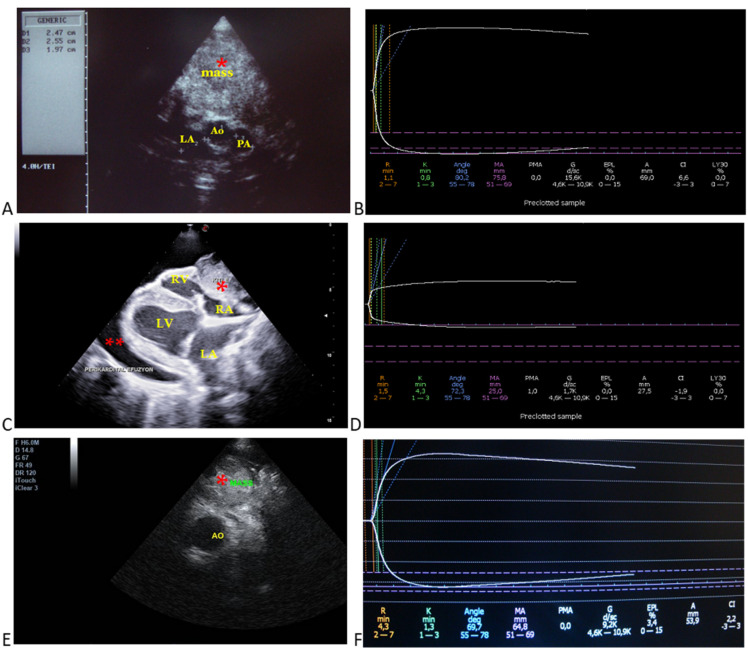
Selected echocardiographic images and respective thromboelastographic platelet mapping in dogs with cardiac tumors. (**A**) (Case 2): aortic body tumor in a golden retriever; aortic (Ao: 2.4 cm), main pulmonary artery (PA: 1.9 cm), and left atrial diameters (LA: 2.5 cm) from RPSAx view—aortic level, tumor/mass attached to the aorta. (**B**) respective TEG profile indicating hypercoagulable state based on the shortened R and K times, increased alpha angle, MA, and G values. (**C**) (Case 10): pericardial mesothelioma attached to the right atrium and ventricle with pericardial effusion from RPLAx view. (**D**) respective TEG profile indicating hypocoagulable state (low quality of clot strength) based on the decreased MA and G values, as well as the decreased A value. (**E**) (Case 8): heart base tumor in a boxer; tumor/mass attached to the aorta from RPSAx view—aortic level. (**F**) respective TEG profile indicating normocoagulable state based on its reference ranges. Case details can be seen in Table 1. *, mass; **, pericardial effusion; Ao, aorta; PA, pulmonary artery; LA, left atrium; LV, left ventricle; RA, right atrium; RV, right ventricle.

**Figure 2:**
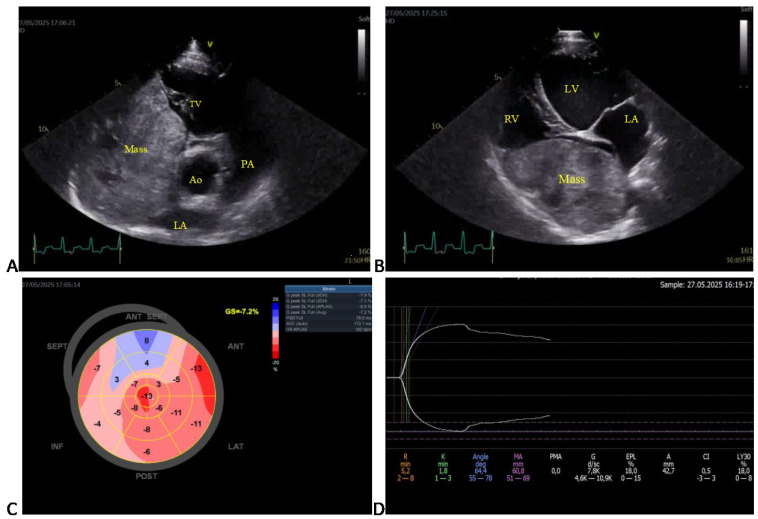
Selected echocardiographic images and corresponding thromboelastographic platelet mapping (TEG-PM) in a large mixed-breed dog with an intracardiac tumor (Case 11). (**A**) Right parasternal short-axis (RPSAx) view at the aortic level showing a large right atrial mass occupying most of the right atrium. (**B**) Apical four-chamber (4Ch) view of the same mass. (**C**) Speckle-tracking echocardiography revealing a markedly reduced global longitudinal strain (GLS = –7.2%; reference less than –17%). (**D**) TEG-PM showing normal reaction (R) and clot formation (K) times, α-angle, and maximum amplitude (MA), but increased projected MA and estimated percent lysis (EPL), suggestive of secondary hyperfibrinolysis. Ao, aorta; LA, left atrium; PA, pulmonary artery; TV, tricuspid valve; LV, left ventricle; RV, right atrium.

**Figure 3:**
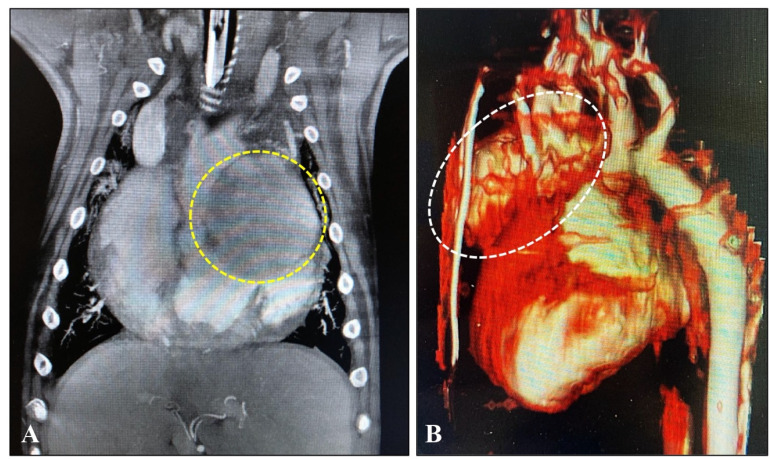
(**A**) MRI: Image of tumor (in a circle) attached to the left atrium of the heart in a golden retriever dog aged 8.5 years the coronary plane on a contrast-enhanced T1 FATSAT image. (**B**) CT: 3D reconstruction image of a tumor (in an ellipse) attached to the ascending aorta in an English bulldog aged 7.6 years (for the dog’s details, please look at Table 1).

**Figure 4:**
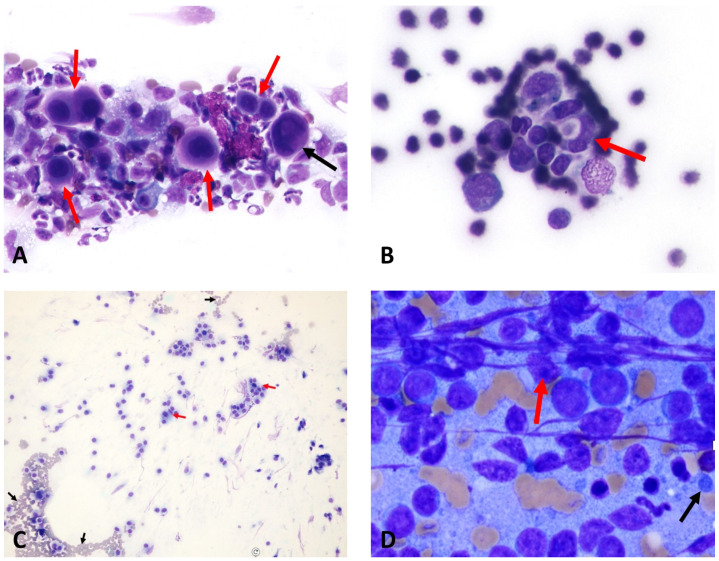
The following images are of cytological samples obtained from patients who underwent fine-needle aspiration biopsy. (**A**) Mesothelioma: Mesothelial cells (red arrows) showing features of binucleation and trinucleation (black arrow), with nuclear pleomorphism and prominent nucleoli (×40 objective) (Case 12). (**B**) Cardiac hemangiosarcoma: Lumen-forming (red arrow), spindle-shaped endothelial cells with basophilic cytoplasm and euchromatic to normochromatic nuclei (×40 objective) (Case 14). (**C**) Aortic body chemodectoma: The presence of eosinophilic granular cytoplasm and round–oval-shaped tumor cells with nuclei (red arrows) is observed, along with erythrocytes (black arrows) in the background (×10 objective) (Case 9). (**D**) Lymphoma: Lymphocytes are observed to be dispersed individually, with a notable presence of numerous distinct nucleoli (red arrow) within the nucleus, and the presence of lymphoglandular bodies (black arrow) is also noted in the background (Case 6). Case details can be seen in Table 1.

### 3.2. Hemato-Biochemical Parameters

No statistically significant differences were detected in the hematologic parameters and serum biochemical markers selected for this study between the groups (Table 2). Hematocrit values were slightly lower in dogs with cardiac tumors, although this difference did not reach statistical significance (median: 34.7% vs. 38.9%, *p* = 0.188).

### 3.3. Echocardiographic Findings

Transthoracic echocardiography revealed the presence of pericardial and/or pleural effusion in seven dogs. Echocardiographic parameters were not significantly different between the CT and HC groups (Table 3). Examples of echocardiographic images from 13 dogs with cardiovascular tumors and only one dog with an intracardiac mass (giant right atrial tumor) are shown in Figure 1 and Figure 2, respectively.

### 3.4. Diagnostic Imaging and Cytological Findings

The cardiovascular tumors of some dogs were further detailed using computed tomography and MRI to determine the mass size and extent (Figure 3). Because of the financial limitations of some owners, the assessment of metastatic spread was performed mainly by abdominal ultrasonography. No definitive metastases were detected in the study population. In one case, cytological findings were suggestive of possible metastatic spread; however, this dog did not exhibit a hypercoagulable state in the TEG analysis.

In the examination of cytological samples obtained from pericardial and/or pleural effusion, the masses were found to be consistent with mesothelioma (Figure 4A), cardiac hemangiosarcoma (Figure 4B), aortic body chemodectoma (Figure 4C), and lymphoma (Figure 4D).

### 3.5. Coagulation Analysis

**Figure 5:**
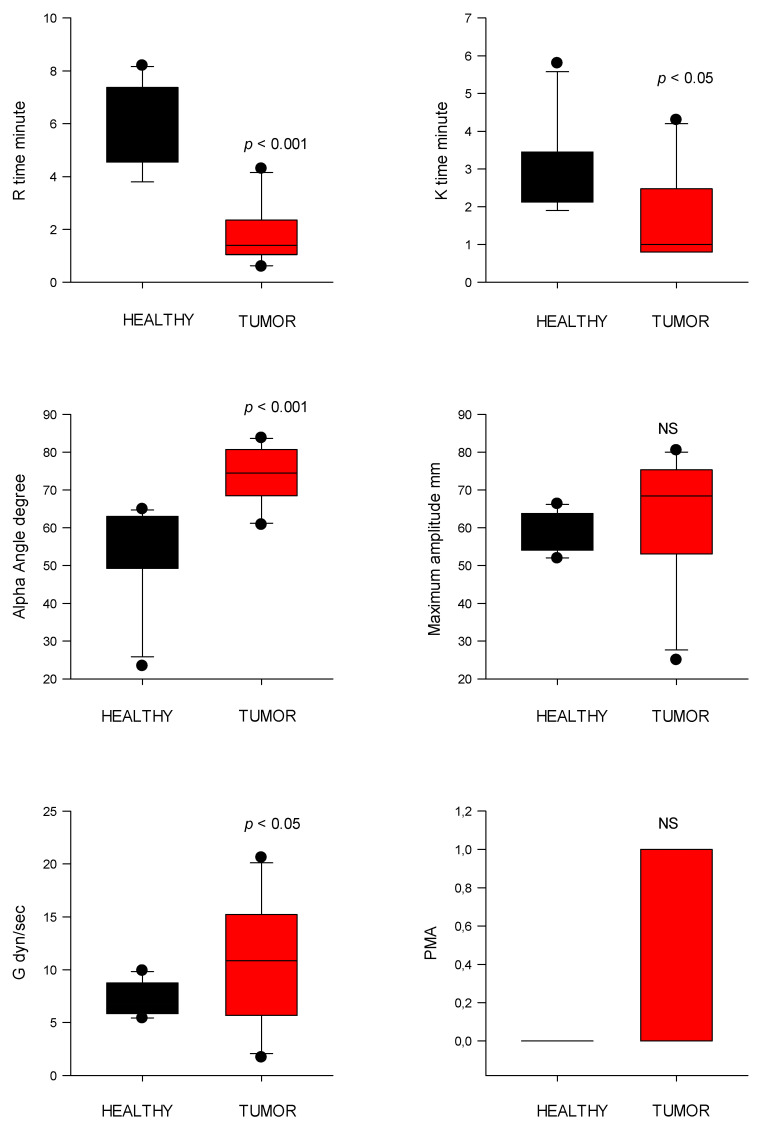
Thromboelastography (TEG) parameters of initial clot formation (R and K time) and clot strength (alpha angle, MA, G, and PMA) from healthy control dogs (healthy) and dogs with cardiac tumors. R, reaction time; K, kinetic time; PMA, projected maximal amplitude; NS indicates non-significance (*p* > 0.05).

**Figure 6:**
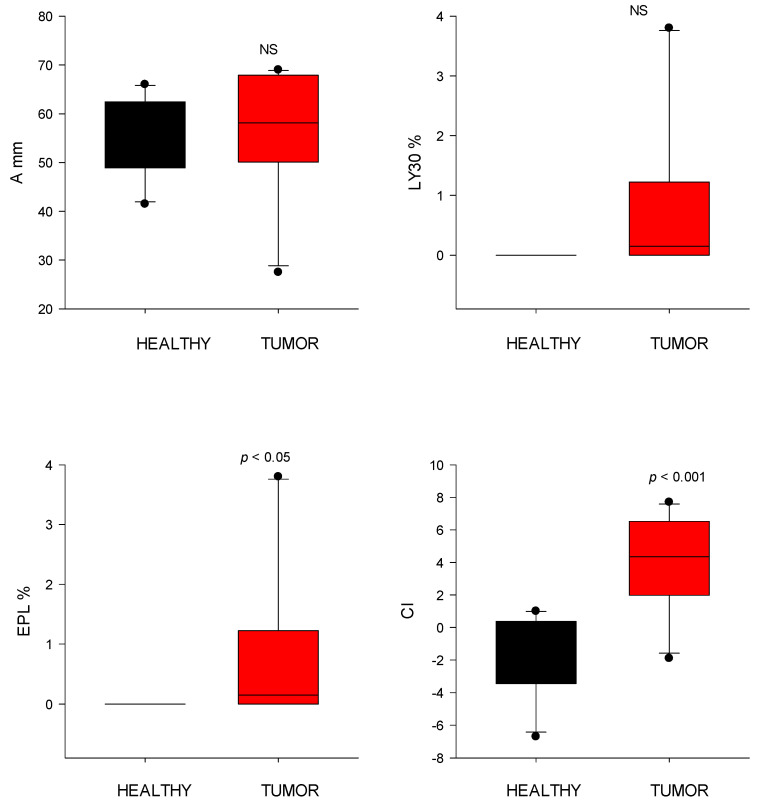
Thromboelastography (TEG) parameters of clot dissolution (fibrinolysis) from healthy control dogs (healthy) and dogs with cardiac tumors. Clot strength (A), clot lysis at 30 min (LY30), estimated percent lysis (EPL), and coagulation index (CI) values. NS indicates non-significance (*p* > 0.05).

TEG platelet mapping showed the presence of hypercoagulation (n = 8), hypocoagulation (n = 3), and normocoagulation (n = 2) in the cardiovascular tumors (Table 1 and Figure 1), as well as secondary hyperfibrinolysis in a dog with an intracardiac tumor (Table 2 and Figure 2). The TEG parameters were presented for initial clot formation and clot strength (Figure 5) and clot dissolution (Figure 6). Additionally, dogs with pericardial and/or pleural hemorrhagic effusions secondary to different types of cardiac tumors exhibited varying coagulation statuses. Three out of four dogs with mesothelioma demonstrated a hypocoagulable state, while the remaining two showed either normocoagulability or hypercoagulability. However, two dogs diagnosed with cardiac hemangiosarcoma were found to be in a hypercoagulable state.

TEG map parameter R time was significantly shorter (*p* < 0.001) in the CT group (2.9 ± 2.7 s) compared to controls (5.9 ± 1.6 s). Similarly, clot formation time (K time) was reduced (*p* < 0.05) in the CT group (1.8 ± 1.3 s) versus controls (2.9 ± 1.1 s). The α-angle, reflecting fibrin build-up rate, in the CT group was significantly higher (69.9 ± 11.8°; *p* < 0.001) compared to controls (53.8 ± 12.3°) (Figure 5). MA was higher in the CT group (63.5 ± 16.1 mm) but not significantly different than that of control dogs (58.2 ± 5.0 mm).

PMA was significantly increased in the CT group (0.38 ± 0.50) versus controls (0.00 ± 0.00; *p* = 0.025). The G value, representing overall clot strength, was also elevated in the CT group (10.8 ± 5.5 dyn/cm^2^) compared to controls (7.2 ± 1.5 dyn/cm^2^; *p* < 0.05) (Figure 5).

EPL did not differ significantly between groups (tumor: 1.0 ± 1.8%; control: 0.00 ± 0.0%). The CI was significantly higher (*p* < 0.001) in tumor-bearing dogs (4.0 ± 2.9) compared to controls (−1.5 ± 2.5), consistent with a hypercoagulable state (Figure 6).

**Figure 7:**
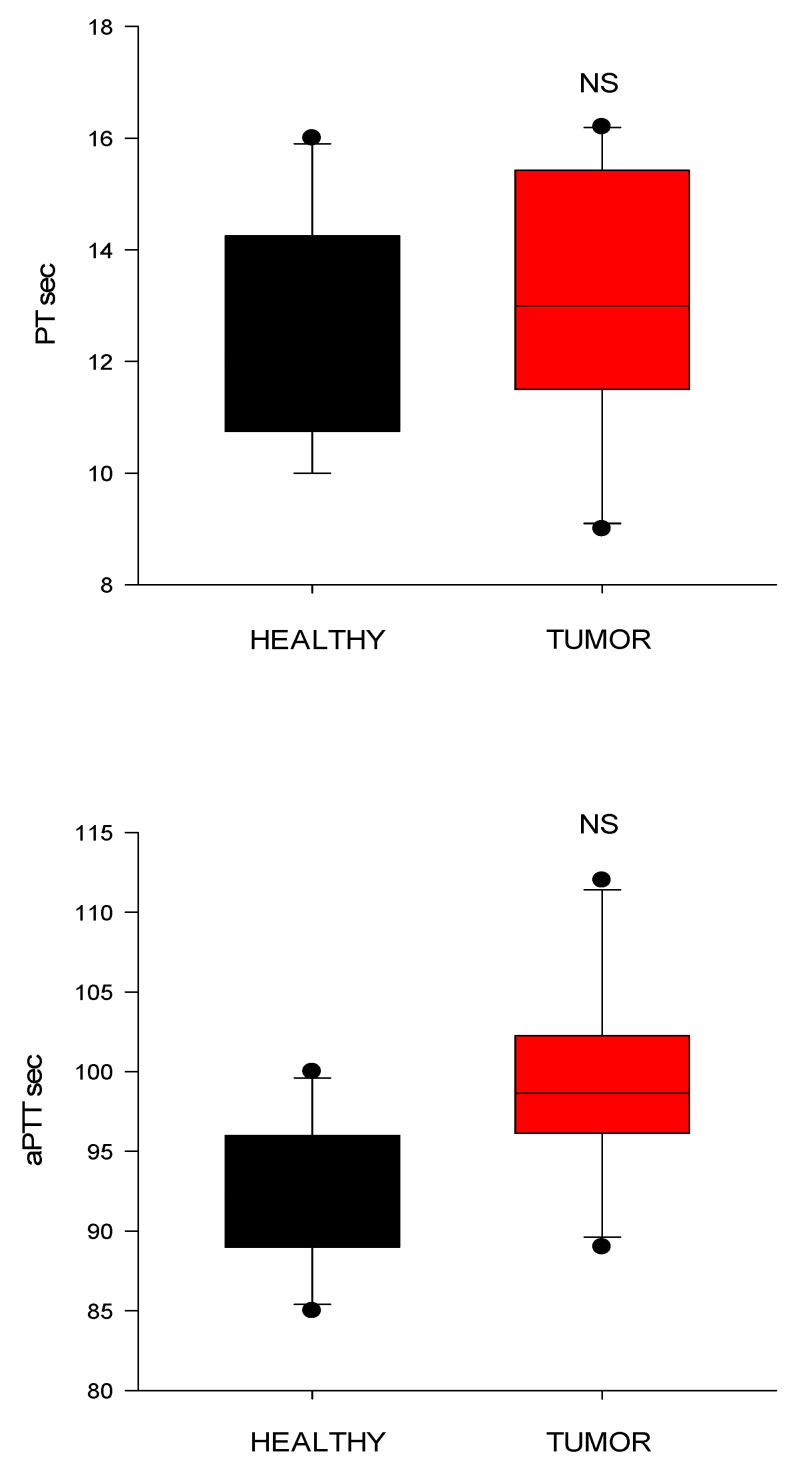
Conventional clotting times from healthy control dogs (healthy) and dogs with cardiac tumors. PT, prothrombin time; aPTT, activated partial thromboplastin time (CI) values; NS indicates non-significance (*p* > 0.05).

Conventional coagulation tests (PT and aPTT) showed no significant differences between the groups (Figure 7). Both of them tended to increase slightly (for PT, *p* = 0.09; for aPTT, *p* = 0.06) in dogs with tumors than in controls.

### 3.6. Correlation Analysis

Correlation analysis revealed no statistically significant associations between TEG variables and clinical, hematological, or echocardiographic parameters (Table 4). However, a significant negative correlation was observed between EPL and both PT (*r* = −0.739, *p* = 0.009) and cardiac EF (*r* = −0.737, *p* = 0.009). Additionally, moderate but non-significant correlations were noted between the α-angle and LA/Ao ratio (*r* = −0.425, *p* = 0.220), and between CI and LA/Ao ratio (*r* = −0.504, *p* = 0.137). The other TEG parameters, including R-time, K-time, MA, G, and LY30, showed no meaningful correlations with the evaluated variables.

## 4. Discussion

This study focused primarily on evaluating the coagulation status of dogs with cardiac tumors using TEG, rather than detailing tumor identification or histopathologic classification procedures. Our findings suggest that hypercoagulability may be a predominant feature in dogs with cardiac tumors; however, there is considerable variability in coagulation status among individual cases, preventing generalization. These observations from dogs with various types of cardiac tumors are consistent with the prothrombotic tendencies reported in cancer-bearing individuals, particularly those with cardiovascular involvement, in human oncology [1,4,5].

Cardiac tumors can present with a wide range of clinical signs, from mild to severe and life-threatening, or may be discovered incidentally, particularly in large-breed and aged dogs [1,5,30], as demonstrated in this study. The clinical manifestations are often independent of the histological type and are typically related to compromised cardiovascular function due to the mass effect [1]. Dogs in the CT group demonstrated significantly increased heart and respiratory rates, likely reflecting hemodynamic and systemic stress associated with the presence of space-occupying lesions in or around the heart [2,28]. Among the dogs with cardiac tumors, pericardial and/or pleural effusion was observed in seven cases and was accompanied by higher heart and respiratory rates. However, these changes were not statistically significant, suggesting that while effusion may contribute to hemodynamic stress, it is not a sole determinant of elevated vital parameters. In addition, three dogs were documented to be hypertensive, which may further reflect hemodynamic stress and systemic cardiovascular compromise associated with cardiac tumors. Although the number of cases was limited, this observation supports the notion that cardiac neoplasia can contribute to elevated afterload and blood pressure dysregulation.

Although they were not statistically significant, the echocardiographic findings suggested trends toward increased median left atrial size and left ventricular internal diameter, along with reduced fractional shortening and global longitudinal strain rate (notably in the dog with a right atrial mass). These patterns may indicate early cardiac remodeling and subclinical systolic dysfunction, potentially secondary to mechanical compression caused by heart base or pericardial tumors [29].

The hypercoagulable state was most clearly detected using TEG in 8 out of the 14 dogs. Dogs with cardiac tumors showed significantly shortened R and K times, alongside elevated α-angles, all indicative of accelerated thrombin generation and faster fibrin polymerization. These changes reflect enhancement of both the initiation and amplification phases of coagulation, likely driven by tumor-associated procoagulant factors, such as tissue factor expression and subsequent activation of factor VII [31] and inflammatory cytokines [32], in dogs with cardiac tumors [33]. Additionally, elevated platelet contribution to clot formation, as evidenced by increased PMA and G values, further supports the presence of enhanced clot stability and platelet–fibrin interaction in the CT group [13].

Although MA was elevated in tumor-bearing dogs, it did not reach statistical significance. EPL was comparable between the groups, suggesting no overt upregulation of fibrinolysis. However, considering the primary hyperfibrinolysis in dogs with sarcomas [34], the presence of secondary hyperfibrinolysis in a dog with an intracardiac mass in this study highlights the potential for heterogeneity in hemostatic responses depending on tumor location and type.

These findings align with prior studies that report increased hypercoagulability in tumor-bearing dogs, with 45–66% of cases showing TEG-based hypercoagulability across various tumor types [24,35,36]. In our cohort, the assessment of metastatic spread was mainly performed by abdominal ultrasonography due to financial limitations, and no definitive metastases were detected. Interestingly, one dog with cytological findings suggestive of metastatic spread did not exhibit a hypercoagulable state according to TEG, further underscoring the heterogeneity of coagulation responses among tumor-bearing dogs and suggesting that metastatic potential alone may not dictate hemostatic alterations. Importantly, the majority of tumors in this cohort were heart-associated but extracardiac in origin, namely, heart base tumors, aortic body tumors, and pericardial mesotheliomas, rather than primarily intracardiac neoplasms. These tumors, while not located within cardiac chambers, can still influence cardiovascular physiology and hemostasis through indirect mechanisms, including pericardial effusion, external cardiac compression, inflammation, and endothelial activation [10].

The TEG results in this population showed clear signs of hypercoagulability (e.g., reduced R time and increased α-angle and G values), likely reflecting these systemic and local prothrombotic effects. However, their extracardiac localization may explain the lack of significant derangements in conventional coagulation parameters (PT and aPTT), as they are less likely to cause direct endocardial injury or turbulent intracardiac flow, key components of Virchow’s triad that are more typically associated with intracardiac masses [9,10]. By contrast, intracardiac tumors, especially those involving the atrial or ventricular endocardium, can promote thrombus formation more directly through flow turbulence, local platelet activation, and endocardial disruption [37]. The limited representation of such tumors in this study may have contributed to the absence of overt thrombosis or abnormalities in standard coagulation profiles.

The heterogeneity in both tumor type and anatomical localization among cardiac tumors in our cohort likely contributes to the variability observed in coagulation profiles. Different tumor types are known to influence the hemostatic system in distinct ways, as evidenced by previous studies reporting significant variation in coagulation abnormalities across malignancies [37]. Cardiac hemangiosarcoma, for example, has been associated with hypercoagulability, thrombocytopenia, and DIC, affecting up to 50% and 75% of dogs, respectively [38], possibly due to high tissue factor expression [39,40]. While coagulation data on chemodectomas are lacking, their proximity to major vessels may induce hypercoagulability via endothelial activation and altered flow dynamics. Similarly, mesotheliomas and lymphomas may exert variable procoagulant effects depending on their invasiveness and inflammatory potential [41]. These findings highlight the need for future studies with larger, stratified cohorts to clarify tumor-specific mechanisms underlying coagulation disturbances [41].

While most correlations between TEG indices and clinical or echocardiographic variables were non-significant, a notable negative correlation was identified between EPL and both PT and EF. In our cohort, this suggests a potential link between reduced systolic function and altered fibrinolytic regulation. However, the clinical meaning of this association remains uncertain. In human heart failure, reports are conflicting: Some studies describe enhanced platelet activation, thrombin generation, and increased fibrinolytic activity in patients with reduced EF [42], while others demonstrate impaired clot structure and a tendency toward prolonged fibrinolysis that correlates with left atrial enlargement and inflammatory biomarkers rather than with EF% itself [43]. In veterinary cardiology, dogs with severe systolic dysfunction such as DCM do not consistently show fibrinolytic abnormalities, underscoring the complexity of this relationship [16]. Moderate, though non-significant, associations between the LA/Ao ratio and both α-angle and CI in our study also support the concept that structural cardiac changes may contribute to prothrombotic tendencies through blood stasis and altered shear forces. This relationship is likely multifactorial, involving blood flow stasis, endothelial dysfunction, and inflammation secondary to cardiac remodeling [44,45], which together enhance hypercoagulable states or thrombotic risk [10,46]. Similar findings have been reported in feline arterial thromboembolism, where viscoelastic parameters demonstrated a hypercoagulable state [47]. Therefore, our observation should be regarded as a hypothesis-generating finding and interpreted with caution, rather than as a definitive causal link.

Conventional coagulation tests (PT and aPTT) failed to identify coagulation disturbances in this cohort, as previously reported; their limited sensitivity for detecting hypercoagulability is underscored by the fact that they assess only isolated components of the coagulation cascade [14,20]. In contrast, TEG offers a comprehensive assessment encompassing clot formation kinetics, strength, and stability, as well as fibrinolytic activity—parameters more reflective of in vivo hemostatic potential [14,20].

This study has several limitations. Due to restricted funding, comprehensive biomarker analysis and post-treatment evaluations could not be performed uniformly across all patients. Follow-up assessments and certain diagnostic tests were reliant on caregivers, leading to inconsistent data collection. Additionally, the lack of histopathological confirmation in many cases is a limitation of this study, mainly due to patient reluctance to consent to necropsy and challenges with long-term follow-up. Consequently, tumor diagnoses were based on imaging techniques such as transthoracic echocardiography, CT/MRI, and FNA cytology, which may have reduced diagnostic accuracy, particularly in tumors with poor exfoliative properties [1,20,48]. Given the anatomical risks of intracardiac and intrathoracic masses, FNA was chosen as a minimally invasive approach. FNA has demonstrated a high diagnostic accuracy (92%) when combined with imaging, with sensitivity for neoplasia reaching 91% [49]. While pericardial fluid cytology is often used, its diagnostic yield can be limited, particularly when samples are not obtained directly from the mass [50]. Despite its limitations, FNA remains an essential tool for preliminary tumor evaluation when histopathology is not possible. The small sample size further limits the statistical power and generalizability of the findings. Additionally, in future studies, evaluating the differences in coagulation changes between intracardiac and extracardiac tumors would be valuable. Serial TEG assessments were not performed, preventing the evaluation of dynamic changes in coagulation throughout the disease course or in response to treatment [51]. Furthermore, advanced echocardiographic techniques, including speckle-tracking and left ventricular global strain analysis, were largely unavailable and only utilized in a single case, limiting the assessment of myocardial function. Additionally, although coagulation status may be influenced by factors such as physiological stress [52], diet [53], or subclinical inflammation [54], all coagulation analyses were performed under standardized and uniform conditions by the same personnel, and strict inclusion criteria were applied to reduce potential confounders. Lastly, the absence of specific coagulation parameter measurements, such as individual clotting factors, D-dimer levels, or platelet function testing, hindered a more detailed understanding of hypercoagulability and thromboembolic risk in this population.

## 5. Conclusions

Our study demonstrates that dogs with cardiac tumors, particularly heart-associated extracardiac neoplasms, exhibit a TEG-defined hypercoagulable state characterized by accelerated coagulation initiation, enhanced platelet contribution, and increased clot strength. Although hypercoagulability was the most frequently observed phenotype, the coagulation profiles within our study population were heterogeneous. While some trends regarding correlations between TEG-derived parameters and cardiac chamber dilation were noted, these were not statistically significant. To better elucidate potential relationships between cardiac tumors and cardiac function in dogs, future studies with larger sample sizes and more detailed cardiological evaluations, as reported in human medicine [55], are warranted. These hemostatic alterations occur despite normal results on conventional coagulation assays and may be modulated by tumor location, malignancy, and cardiac function. TEG emerges as a valuable tool for identifying subclinical coagulopathies in canine oncology, underscoring its potential utility in thrombotic risk assessment and anticoagulation decision-making in affected patients.

## Figures and Tables

**Table 1:** Clinical and pathological findings, along with thromboelastographic (TEG) profiles from 14 dogs studied.

Dog	Breed	Age (y)	Sex	BW (kg)	Findings—Predominant	Tumor Type	TEG Profile
1 *	Golden retriever	8.5	F	32.4	Tachypnea, tachycardia, exercise intolerance	Heart base tumor	Hypercoagulable
**2 ^θ^**	Golden retriever	9.3	F	37.2	Tachycardia, tachypnea, coughing	Aortic body tumor	Hypercoagulable
**3**	Golden retriever	7.9	M	33.4	Muffled heart sound, arrhythmias, tachypnea	Aortic body tumor	Hypercoagulable
**4**	Labrador retriever	8.0	M	38.5	Tachycardia, tachypnea, hemorrhagic pericardial effusion	Heart base tumor	Hypocoagulable
**5**	Labrador retriever	9.1	F	37.1	Weakness, pleural and pericardial effusion	Pericardial mesothelioma	Hypercoagulable
**6 ^π^**	German shepherd	8.2	M	38.4	Exercise intolerance, tachycardia, tachypnea	Lymphoma	Hypercoagulable
**7**	German shepherd	5.4	M	34.4	Weakness, syncope, pericardial effusion	Pericardial mesothelioma	Normocoagulable
**8 ^θ^**	Boxer	8.4	F	36.4	Lethargy, ascites, tachycardia	Heart base tumor	Normocoagulable
9 **^π^	English bulldog	7.6	M	29.3	Exercise intolerance, inappetence, tachypnea	Aortic body tumor (chemodectoma)	Hypercoagulable
**10 ^θ^**	Mixed breed	8.6	M	46.2	Exercise intolerance, ascites, pericardial and pleural effusion	Pericardial mesothelioma	Hypocoagulable
**11**	Mixed breed	9.2	M	45.3	Lethargy, jugular distention, ascites	Right atrial tumor unclassified	Seconder hyperfibrinolysis
**12 ^π^**	Golden retriever	8.0	M	27.2	Exercise intolerance, ascites, pericardial and pleural effusion	Pericardial mesothelioma	Hypocoagulable
**13**	Golden retriever	9.0	F	23.3	Tachycardia, tachypnea, exercise intolarance	Cardiac hemangiosarcomas	Hypercoagulable
**14 ^π^**	Cane corso	8.4	F	35.2	Weight loss, coughing, pleural effusion	Cardiac hemangiosarcomas	Hypercoagulable

The following diagnostic tools and findings are presented in the figures: echocardiographic localization of cardiac tumors with their corresponding thromboelastographic images (^θ^ Figure 1 and Figure 2), diagnostic (* MRI and ** CT) imaging (Figure 3), and fine-needle aspiration cytology (^π^ FNAC) (Figure 4). y, years; M, male; F, female; BW, body weight.

**Table 2:** Signalment and selected clinical and serum biochemistry findings in healthy (control) dogs and dogs with cardiac tumors.

Variable	Control (n = 10)		Cardiac Tumor (n = 14)		*p*-Value
	Median	Min–Max	Median	Min–Max	
**Signalment**					
**Age (year)**	8.5	6.1–9.2	8.2	5.4–9.3	0.73
**Body weight (kg)**	40.2	32.4–48.0	34.2	23.0–46.2	0.68
**Heart rate (beat/min)**	100	88–112	118	90–160	0.017
**Respiratory rate (breath/min)**	16	12–20	42	12–104	0.001
**Body temperature (°C)**	38.1	37.8–38.5	38.7	38.3–39.6	0.876
**Hemogram**					
**White blood cells (×10^3^/μL)**	14.1	10.4–14.6	22.1	10.9–33.3	0.422
**Hematocrit (%)**	38.9	35.0–44.6	34.7	25.7–47.9	0.188
**Platelets (×10^6^/μL)**	316	254–412	336	182–464	0.157
**Serum biochemistry**					
**Serum total protein g/dL**	7.5	6.6–8.1	7.5	6.5–7.9	0.887
**ALP IU**	45.3	35.3–67.2	174.8	19–460	0.874
**ALT**	24.0	21.3–56.2	42.9	34.1–45.9	0.758
**BUN g/dL**	16.00	6.2–22.0	27.3	11.0–38.3	0.754
**Cr g/dL**	0.6	0.3–0.8	0.8	0.5–0.9	0.544

Min, minimum value; Max, maximum value.

**Table 3:** Selected echocardiographic parameters in healthy (control) dogs and dogs with cardiac tumors.

Variable	Control (n = 10)		Cardiac Tumor (n = 14)		*p*-Value
	Median	Min–Max	Median	Min–Max	
**LA/Ao ratio**	1.1	1.0–1.3	1.4	1.0–1.6	0.088
**LVIDdN**	1.3	1.2–1.4	1.4	1.3–1.7	0.062
**FS%**	39	33–42	34	12–43	0.053
**EF%**	69	62–75	65	55–73	0.541

**Table 4:** Correlation study between thromboelastography variables and some clinical, hematological, and serum biochemical parameters.

TEG	PT	aPTT	EF	FS	LA/Ao	LVIDdN
**R time**	*r*: 0.100 *p*: 0.783	0.120 0.786	−0.484 0.156	−0.270 0.450	−0.028 0.939	0.309 0.386
**K time**	*r*: 0.07 *p*: 0.837	0.17 0.719	0.23 0.639	0.285 0.390	0.220 0.542	0.378 0.282
**α-angle**	*r*: −0.291 *p*: 0.415	−0.393 0.230	0.071 0.844	0.315 0.340	0.045 0.900	−0.425 0.220
**MA**	*r*: −0.115 *p*: 0.752	−0.177 0.600	−0.051 0.888	−0.027 0.93	0.096 0.791	−0.422 0.225
**PMA**	*r*: 0.169 *p*: 0.31	−0.167 0.610	0.368 0.270	−0.027 0.93	−0.200 0.579	0.294 0.409
**G**	*r*: −0.143 *p*: 0.694	−0.081 0.81	0.061 0.867	0.144 0.67	0.036 0.921	−0.399 0.254
**EPL**	*r*: −0.012 *p*: 0.742	−0.739 0.009	−0.083 0.818	−0.737 0.009	−0.048 0.894	0.030 0.934
**A**	*r*: −0.178 *p*: 0.622	−0.174 0.600	−0.269 0.42	−0.101 0.76	0.074 0.838	−0.422 0.224
**CI**	*r*: −0.160 *p*: 0.660	−0.409 0.21	0.060 0.869	0.325 0.33	0.104 0.775	−0.504 0.137
**LY30**	*r*: −0.120 *p*: 0.742	−0.305 0.360	−0.277 0.439	−0.495 0.12	−0.048 0.894	0.030 0.934

## Data Availability

Original data are available from first author (Z.Y.) upon request.

## Abbreviations

The following abbreviations are used in this manuscript:

PT	Prothrombin Time
aPTT	Activated Partial Thromboplastin Time
TEG	Thromboelastography
CT	Computed Tomography
MRI	Magnetic Resonance Imaging
CBC	Complete Blood Count
ALT	Alanine Aminotransferase
ALP	Alkaline Phosphatase
BUN	Blood Urea Nitrogen
R	Reaction Time
K	Clot Kinetics
MA	Maximum Amplitude
G	Clot Strength
PMA	Projected Maximum Amplitude
EPL	Estimated Percent Lysis
LY30	Lysis at 30 Minutes
CI	Coagulation Index
RPSAx	Right Parasternal Short Axis
RPLAx	Right Parasternal Long Axis
LA/Ao	Left Atrium-to-Aorta Ratio
LVIDdN	Left Ventricular Internal Diameter Normalized for Body Weight
FS%	Fractional Shortening
EF%	Ejection Fraction
GLS%	Global Longitudinal Strain
SD	Standard Deviation
